# The Long-Term Characteristics of Immunity Conferred by COVID-19 Using Antibody Tests

**DOI:** 10.7759/cureus.17731

**Published:** 2021-09-05

**Authors:** Faryal Shoaib, Muhammad Ashraf, Hashaam Ghafoor, Imran N Ahmad, Ghazanfar Abbas

**Affiliations:** 1 Internal Medicine, Shifa International Hospital Islamabad, Islamabad, PAK; 2 Anesthesia, Hamad Medical Corporation, Al Khor, QAT; 3 Pathology, Shifa International Hospital Islamabad, Islamabad, PAK; 4 Chemical Pathology, Shifa International Hospital Islamabad, Islamabad, PAK

**Keywords:** reinfection, comorbities, covid-19, anti-sars cov2 antibody, immunity, healthcare workers

## Abstract

Introduction

Since December 2019, more than 184 Million cases and 3.97 Million COVID-19 related deaths have been reported around the world. Since these statistics are laboratory-based confirmed cases, the true burden of disease may be underestimated. Many populations like those who are regularly visiting health care facilities and those with end-stage renal disease (ESRD) for visiting dialysis units, patients with malignancies, on regular chemo and radiotherapy, and healthcare workers (HCW) are considered high risk for nosocomial COVID-19 re-infections.

Objective

To understand the long-term behaviour and protective efficacy of already formed anti-SARS-CoV-2 antibody against spike-S protein and nucleocapsid antigen in different populations keeping in view their risk of re-exposure and re-infection is high. To delineate seropositivity with respect to age, gender, and other co-morbidities like diabetes mellitus (DM), hypertension (HTN), and chronic kidney disease (CKD)/ESRD as well as the general population.

Methodology

During the study, 480 cases of COVID-19 with a post-exposure antibody reactive were followed. These patients were followed on telemedicine for the development of reinfection symptoms and persistence of antibody response. Around 115 patients agreed for regular monitoring of their immunity against the COVID-19 virus through testing through the anti-SARS-CoV-2 antibody test. The rest of the patients were followed on telemedicine until the date of development of any re-infection, but none reported to have typical symptoms of COVID-19 along with positive polymerase chain reaction (PCR).

Results

Among 115 patients, the mean age was 42.44 + 15.755 years. 61.7% of patients were males and 66.1% were non-health workers while 26.1% of patients had DM/HTN or both. Among these patients, 76.5% had mild/no symptoms and antibodies were found present among 51.3% patients for 3-6 months. Only 2.6% of patients were re-infected. Significant association (p<0.05) of age was found with re-infection while insignificant association (p>0.05) of sex, co-morbidities, profession, symptoms, and persistence of antibodies with re-infection.

Conclusion

The study concluded that natural immune response was adequate to protect against reinfection as long as more than 9 months. It was more pronounced among patients with ESRD and those with severe disease. Surprisingly, among patients with haematological malignancies, either there was no seropositivity or a very weak positive antibody response. All other malignancies had similar seropositivity behaviour compared to the general population or other co-morbidity like DM, HTN, and coronary artery disease (CAD).

## Introduction

In the month of December 2019, a novel coronavirus, emerged in Wuhan, China that caused SARS-CoV-2 infection (severe acute respiratory syndrome). Since then, the SARS-CoV-2 virus (severe acute respiratory syndrome coronavirus 2) has raised a pandemic involving almost all countries [1,2]. The World Health Organisation (WHO) subsequently named the disease caused by SARS‐CoV‐2 as COVID‐19 (coronavirus disease) on February 11, 2020. The virus spread rapidly and the World Health Organisation declared it a global pandemic [3].

The COVID-19 can be transmitted from one person to other through breathing in of the respiratory droplets from the infected individuals and through direct contact with infected surfaces and objects [4]. Mostly, mild symptoms have been developed among COVID-19 several patients, for instance, fever, dry cough, and sore throat. The majority of these patients have recovered while several patients infected with coronavirus have been confirmed to be entirely asymptomatic. Though, some patients have developed several lethal complications such as organ failure, pulmonary edema, septic shock, atypical acute respiratory distress syndrome, and severe pneumonia. Normally, the patients who need ICU admission are older (age more than 60 years) or with multiple co-morbidities namely endocrine, cardiovascular, digestive, respiratory, and cerebrovascular diseases [5].

Considering the huge clinical, social, financial, and psychological impact of this novel outbreak, it is extremely crucial to examine the possible responses of the human immune system during SARS-CoV-2 infectivity and the role of virus-specific T cells and B-lymphocytes. As in all viral infections, adaptive immune responses are mediated by virus-specific T cells, and cell-mediated immunity by B-lymphocytes, for humoral immunity, play an important role [6].

SARS-CoV-2 belongs to the *Betacoronavirus *type in the family *Coronaviridae *that offers four main antigenic proteins: spike (S), envelope (E), membrane (M), and nucleocapsid (N). The S protein is made of S1 and S2 subunits, while S1 is major binding protein between host cell receptors and virus. The RBD (receptor-binding domain) in S1 subunit interacts with human cells which express ACE2 (angiotensin-converting enzyme 2) and facilitate virus entry [7]. Regarding the adaptive immune responses during coronavirus disease 2019, both cellular and humoral immune responses to the SARS-CoV-2 are crucial regarding anti-infection activities. Humoral immune responses to the SARS-CoV-2 are mediated through antibodies directly targeting the viral surface glycoproteins, primarily the spike glycoprotein and nucleocapsid protein [1]. The 180 kDa spike glycoprotein holds two subunits (i.e. C-terminal S2 and N-terminal S1) and is believed to be a significant antigenic determinant that is able to induce a protective immune response [8]. The S1 subunit has a receptor-binding domain (RBD; residues 331-524), that mediates viral binding to functional ACE2 receptors on vulnerable cells and is the major target for the SARS-CoV-2 neutralising antibodies [9].

Antibodies specific to COVID -19, which are generated by either vaccination, infectivity, or both (anti-spike glycoprotein and anti-receptor-binding domain) are considered significant for the neutralisation and clearance of COVID- 19 virus and are quantified by in vitro neutralisation assays [1]. There is scarcity of data regarding post-infectivity immunity to the SARS-CoV-2, and genetic and biological factors which are held responsible for a broad range of disease acuteness remain vague [1]. Therefore, antibody titres could be good biomarkers regarding antibodies protective efficacy as well as successful humoral immune responses after the severe acute respiratory syndrome coronavirus 2 exposure [10]. Several studies have reported serological investigative tests utilizing nucleocapsid and/or spike protein from severe acute respiratory syndrome coronavirus 2 by enzyme-linked immunosorbent assay (ELISA) [11,12,13], immunofluorescence [14] and even a lateral flow test [15].

## Materials and methods

After approval by the Institutional Review Board and Ethics Committee (IRB & EC), Shifa International Hospital Ltd (SIH), in this uni center, prospective observational study, we followed 480 patients from inpatient and out-patient department (OPD) of SIH, with confirmed COVID-19 infection who had initial antibody tests done from May 2020 to July 2020. These patients were kept under surveillance through teleconsultation and telephonic survey, for the development of any reinfection, and their antibody responses were monitored through repeat testing as per their convenience. Only 115 random patients agreed for repeat testing over a period of more than 10 months; the rest of the patients were followed up on telephone for development of reinfection till date. None of these 115 patients had received vaccination as this study was done before the vaccination era in Pakistan. 

We defined a patient with a mild disease as having only fever and respiratory or gastrointestinal symptoms without the requirement of supplementary oxygen. Moderate disease was defined as symptoms of COVID-19 along with the requirement of supplementary oxygen and those with severe disease required ICU/HDU (high dependency unit) admission and/or requirement of ventilatory support.

We used two methods to check serological response: the first test was used to determine antibody against nucleocapsid antigen and was a qualitative method, and the second method was used to determine antibody against spike S protein and it was a quantitative method. The prevalence of COVID-19 in Islamabad was 14.7% in July 2020, as per the National Institute of Health (NIH), Islamabad.

Qualitative method

In this method, anti-SARS-CoV-2, which is an immunoassay for the in vitro qualitative detection of antibodies and includes IgG and IgM in human serum and plasma against nucleocapsid antigen. It is analyzed on Roche cobas (Roche, Basel, Switzerland) based on the electrochemiluminescence principle. Serological assay which detects antibodies against SARS-CoV-2 can contribute to identifying individuals with a previous infection and assessing the extent of exposure. Here, those with values of up to 40 and above the cut-off index >1.0 were considered weak reactive/positive and those with value >80 cut-off index were considered strongly reactive/positive. It showed only strong or weak positivity/reactivity, not actual titers of antibody.

Reagent and working solution included are M, R1, and R2. M contains streptavidin-coated microparticle while R1 has a specific recombinant antigen (*E. coli*) and buffer and R2 contain antigen with ruthenium complex, and all have specific pH that is 7.7.

Specimen Collection: Lithium heparin, K2-EDTA (dipotassium ethylenediaminetetraacetic acid), and K3-EDTA sampling tube.

Test Principle: The test principle is the sandwich immunoassay in which streptavidin microparticle is used.

Table 1 shows the type of reaction, incubation, and procedure.

**Table 1:** The qualitative method

1^st^ Incubation	20 µL sample and severe acute respiratory syndrome coronavirus 2 specific recombinant antigen tagged with ruthenium complex.	Sandwich complex formed.
2^nd^ incubation	Streptovidin-coated microparticle and complex bound to solid phase through interaction with biotin and streptavidin.	
Reaction mixture	Aspiration in measuring the cell in which microparticles are captured magnetically onto the surface.	Unbound substance removed with pro cell/pro cell M.
Electrical activity	Application of voltage to electrode then produced chemiluminescent emission.	Measured reaction by a photomultiplier and get signals from the reaction product.
Cutt off	<1.0 >1.0	Non-reactive (negative) Reactive (positive)
Specificity	99.5%	

Quantitative method

Anti-severe acute respiratory syndrome coronavirus-2 S proteins is an immunoassay for in vitro quantitative determination of antibodies against SARS CoV-2 and detect S protein RBD in human plasma and serum and it includes IgG and IgM antibody titers in human serum and plasma. It is an immunoassay with the purpose to assess the adoptive humoral response to the severe acute respiratory syndrome coronavirus 2 S protein and is helpful in monitoring the dynamics of antibody response in individual patients. It is analyzed on Roche Cobas based on the electrochemiluminescence principle. A serological assay that identifies antibodies against severe acute respiratory syndrome coronavirus 2 can contribute to detecting individuals with a previous infection and assessing the extent of exposure.

Reagent and working solution included are M, R1, and R2. M contains streptavidin-coated microparticles while R1 has severe acute respiratory syndrome coronavirus 2 S Ag biotinylated receptor-binding domain as specific recombinant antigen and buffer, and R2 contains antigen with ruthenium complex, and all have a specific pH that is 7.4.

Traceability: The method is standardizing against the internal Roche standards for anti-SARS-CoV-2 S. It is a mixture of monoclonal antibodies that bind spike 1 RBD at two different epitopes.

Calibration: Calibration should be carried out once per reagent lot utilizing fresh reagent and review calibration if there is same reagent lot after 31 days and after 14 days on same reagent kit or as required.

Specimen Collection: Lithium heparin, K2 EDTA, and sodium citrate.

Test Principle: The test principle is the sandwich immunoassay in which streptavidin microparticles are used.

Table a shows the type of reaction, incubation, and procedure.

**Table 2:** The quantitative method RBD: receptor-binding domain

1^st^ Incubation	20 µL sample and biotinylated SARS-CoV-2 S receptor-binding domain recombinant antigen and SARS-COVID-2 S RBD labeled with ruthenium complex.	Sandwich complex formed.
2^nd^ incubation	Streptavidin-coated microparticle and complex bound to solid phase via interaction with biotin and streptavidin.	
Reaction mixture	Aspiration in measuring cell where microparticles are magnetically captured on to surface.	Unbound substance removed with pro cell/pro cell M.
Electrical activity	Application of voltage to electrode then produced chemiluminescent emission	Measured reaction by photomultiplier and get signals from reaction product.
Cutoff value	<0.8 U/mL >0.8U/mL	Negative Positive
Sensitivity & specificity	98.8% and 99.1%	

Here, the total antibody titer was determined with a maximum value of >250.

It is important to mention that in the qualitative method, antibody presence was detected against nucleocapsid antigen of the COVID -19 virus and incubated at a pH of 7.7, while in the quantitative method, the total antibody titers were detected using pH 7.4. Both methods used different reagents.

## Results

Table 3 describes that among 115 COVID-19 patients, 35 (30.4%) were upto 30 years old and 44 (38.0%) were 31-50 years old, while 36 (31.6%) patients were above 50 years old. The mean age of the patients was 42.44 ± 15.755 years.

**Table 3:** Frequency distribution of COVID-19 patients according to socio-demographic characteristics

	Frequency	%age
Age (years)		
<30	35	30.4
31-50	44	38.0
>50	36	31.6
Total	115	100.0
Mean ± SD	42.44 ± 15.755	
Sex		
Male	71	61.7
Female	44	38.3
Total	115	100.0
Profession		
Health worker	39	33.9
Non health worker	76	66.1
Total	115	100.0

Result shows that out of 115 COVID-19 patients, 71 (61.7%) were males and 44 (38.3%) were females. Among these patients, 39 (33.9%) were health care workers while most of them (76, 66.1%) were non-health workers.

Table 4 depicts that among 115 COVID patients, the majority 52 (45.2%) had no co-morbidity while 30 (26.1%) patients had diabetes mellitus (DM) or hypertension (HTN) or both, followed by chronic kidney disease (CKD)/ end-stage renal disease (ESRD) 14 (12.2%), malignancy 10 (8.7%), and coronary artery disease (CAD) 9 (7.8%).

**Table 4:** Frequency distribution of COVID-19 patients according to co-morbidities DM: Diabetes Mellitus, HTN: Hypertension, CKD: Chronic Kidney Disease, ESRD: End-Stage Renal Disease, CAD: Coronary Artery Disease

	Frequency	%age
DM/HTN or Both	30	26.1
CKD/ESRD	14	12.2
Malignancy	10	8.7
CAD	9	7.8
No co-morbidity	52	45.2
Total	115	100.0

Table 5 presents the symptoms and presence of antibodies among COVID-19 patients and found that out of 115 patients, the majority 88 (76.5%) had mild/no symptoms of the disease, 20 (17.4%) had moderate symptoms, while 7 (6.1%) patients had severe symptoms of the disease.

**Table 5:** Frequency distribution of COVID-19 patients according to symptoms and persistence of antibodies

	Frequency	%age
Symptoms		
Mild/No symptoms	88	76.5
Moderate	20	17.4
Severe	7	6.1
Total	115	100.0
Persistence of antibodies		
<3 months	7	6.1
3-6 months	40	34.8
6-9 months	59	51.3
>9 months	9	7.8
Total	115	100.0

The result shows that antibodies were found present in 7 (6.1%) patients for <3 months after infection while among 40 (34.8%), 59 (51.3%), and 9 (7.8%) patients, the antibodies were present for 3-6 months, 7-9 months, and >9 months, respectively.

Table 6 exhibits that among 115 COVID-19 patients, only 3 (2.6%) were found re-infected while most of them 112 (97.4%) were not re-infected.

**Table 6:** Frequency distribution of COVID-19 patients according to re-infection

	Frequency	%age
Yes	3	2.6
No	112	97.4
Total	115	100.0
	Frequency	%age
Yes	3	2.6
No	112	97.4
Total	115	100.0

Table 7 shows a significant association (p<0.05) of age with re-infection while insignificant association (p>0.05) of sex, co-morbidities, profession, symptoms, and persistence of antibodies with re-infection.

**Table 7:** Factors associated with re-infection among COVID-19 patients DM: Diabetes Mellitus, HTN: Hypertension, CKD: Chronic Kidney Disease, ESRD: End-Stage Renal Disease, CAD: Coronary Artery Disease

	Re-infection		Total	P-value
	Yes	No		
Age (years)				
<30	0 (0.0%)	35 (30.4%)	35 (30.4%)	0.000
31-50	2 (1.7%)	42 (36.3%)	44 (38.0%)	
>50	1 (0.9%)	35 (30.7%)	36 (31.6%)	
Total	3 (2.6%)	112 (97.4%)	115 (100.0%)	
Sex				
Male	2 (1.7%)	69 (60.0%)	71 (61.7%)	0.859
Female	1 (0.9%)	43 (37.4%)	44 (38.3%)	
Total	3 (2.6%)	112 (97.4%)	115 (100.0%)	
Profession				
Health worker	0 (0.0%)	39 (33.9%)	39 (33.9%)	0.209
Non health worker	3 (2.6%)	73 (63.5%)	76 (66.1%)	
Total	3 (2.6%)	112 (97.4%)	115 (100.0%)	
Co-morbidities				
DM/HTN or Both	1 (0.9%)	29 (25.2%)	30 (26.1%)	0.868
CKD/ESRD	0 (0.0%)	14 (12.2%)	14 (12.2%)	
Malignancy	0 (0.0%)	10 (8.7%)	10 (8.7%)	
CAD	0 (0.0%)	9 (7.8%)	9 (7.8%)	
No co-morbidity	2 (1.7%)	50 (43.5%)	52 (45.2%)	
Total	3 (2.6%)	112 (%) (97.4%)	115 (100.0%)	
Symptoms				
Mild/No symptoms	2 (1.7%)	86 (74.8%)	88 (76.5%)	0.760
Moderate	1 (0.9%)	19 (16.5%)	20 (17.4%)	
Severe	0 (0.0%)	7 (6.1%)	7 (6.1%)	
Total	3 (2.6%)	112 (97.4%)	115 (100.0%)	
Persistence of antibodies				
<3 months	0 (0.0%)	7 (6.1%)	7 (6.1%)	0.677
3-6 months	2 (1.7%)	38 (33.1%)	40 (34.8%)	
6-9 months	1 (0.9%)	58 (50.4%)	59 (51.3%)	
>9 months	0 (0.0%)	9 (7.8%)	9 (7.8%)	
Total	3 (2.6%)	112 (97.4%)	115 (100.0%)	

Table 8 clearly showed a very weak initial antibody response that could not provide sufficient protection. It remained weakly reactive even after COVID-19 reinfection. It is important to mention that patient 2 and patient 3 belonged to the same family and all three patients initially had a mild disease but the severity of COVID-19 reinfection was different in all three patients.

**Table 8:** Assessment of antibody response in re-infection Weak reactive <40; Strong reactive >80 PCR: polymerase chain reaction

	Age of patient	Date of initial antibody test	Initial antibody response	Date of re-infection confirmed by PCR test	Date of antibody test after re-infection	Antibody response after re-infection	Severity of re-infection
Patient no 1	29 years	22/07/2020	6.06	09/12/2020	10/01/2021	25.53	Mild disease
Patient no 2	35 years	27/06/20	1.64	11/11/2020	11/11/2020	9.39	Moderate disease
Patient no 3	54 years	30/10/2020	1.93	11/11/2020	19/11/2020, 27/11/2020, 03/12/2020	Negative, 2.73, 11.92	Severe disease (patient expired)

## Discussion

COVID-19 has become a leading global health problem. Its re-infection can occur among recovered patients. The protective immunity after infection with coronavirus disease 2019 is not yet completely known. Therefore, the current study was carried to know the characteristics of long-term immunity conferred by COVID-19 using antibody tests. To acquire appropriate outcomes, 115 COVID-19 patients were included in the study and found that the majority of the patients (68.4%) were up to 50 years old and the remaining proportion (31.6%) was above 50 years old while the mean age of the patients was 42.44 ± 15.755 years. But the findings of a recent study conducted by To and colleagues (2020) demonstrated that the mean age of the patients was 62.34 years[11].

As far as gender of the patients is concerned, the study disclosed that most of the patients (61.7%) were males while 39.3% were female patients. The results of a study carried out by Zhao and coworkers (2020) exhibited a different scenario and reported that female patients were in majority (51.4%) and 48.6% were male patients [13] 

Health care personnel are at higher risk of acquiring infectious diseases in the hospital. The study disclosed that 33.9% of patients were health workers while 66.1% were non-health workers. A most recent study performed by Alasia and Maduka (2021) confirmed that among patients, only 15.6% were health workers while a significant majority (84.4%) was of non-health workers [16].

During the study, co-morbidities were also assessed among COVID-19 patients. The study found that most of the patients (45.2%) had no co-morbidity while 26.1% of patients had DM/HTN or both, followed by CKD/ESRD (12.2%), malignancy (8.7%), and CAD (7.8%). The findings of our study are almost comparable with a study undertaken by Jin and associates (2020) who reported that more than half (55.6%) of the patients had no co-morbidity while 23.3% of patients had hypertension, followed by diabetes (11.6%), cardiovascular diseases (9.3%) and chronic lung diseases (0.2%) [17]. Another study performed by To and colleagues (2020) indicated that among patients, 26.0% had hypertension while 17.0% had diabetes mellitus [11].

When the symptoms of disease among COVID-19 patients were investigated, it is important to mention that a major proportion (76.5%) of patients had mild/no symptoms, 17.4% had moderate symptoms, and only 6.1% of patients had severe symptoms. The results of a similar study done by Alshukry and teammates (2019) which showed that 39.3% of patients were found with no symptoms while 41.0% had mild/moderate symptoms and 19.7% had severe symptoms [18]. The study further indicated that antibodies were present in 6.1% of patients for <3 months after infection while among 34.8%, 51.3%, and 7.8% of patients the antibodies were present for 3-6 months, 7-9 months, and >9 months, respectively. But Dan and colleagues (2021) confirmed in their study that antibodies were observed among 95.0% of patients up to 6 months after infection [19].

It is significant to mention here that when re-infection among COVID-19 patients was assessed, only 2.6% of patients were found re-infected and all of them had initially very low positive or weakly reactive antibodies ( <40) and remained low at the time of reinfection ( <40). The findings of our study are much better than the study carried out by Muhammad (2020) who asserted that 12.1% of patients were found with re-infection [20]. Another study conducted by Hansen and colleagues (2021) reported that protection against re-infection was 80.5 percent [21].

The study also evaluated the factors associated with re-infection among COVID-19 patients: significant association (p<0.05) of age was found with re-infection while insignificant association (p>0.05) of sex, co-morbidities, profession, symptoms, and persistence of antibodies with re-infection. A similar study conducted by dos Santos and collaborators (2021) showed an insignificant association (p>0.05) of age and sex with re-infection [22]. Zhao and coworkers (2020) reported a significant association (p<0.05) of age with re-infection while an insignificant association (p>0.05) of sex with re-infection [13].

The most important aspect of the study was the behavior of anti-SARS-Cov-2 antibodies among different populations. Significantly when these antibodies were followed over a period of time it was found that among 14 patients with CKD, 10 patients with ESRD were maintained on long-term dialysis. In these patients, 9/10 had the strongest antibody response in terms of antibody titer being >250, which was developed during the first 6 months. Conversely, there were a total of 10 patients of different malignancies and among those, four patients had hematological malignancies, where antibody response was either low or did not develop at all initially and remained low over the period of time. Surprisingly none had developed COVID-19 disease again. It is uncertain how these patients remain protected. It may be due to some other neutralizing antibodies against some other antigens apart from spike protein S and nucleocapsid antigens. Further studies are required to confirm these findings.

## Conclusions

The study concluded that the natural immune response was adequate to protect against reinfection for more than 9 months. It was more pronounced among patients with ESRD and those with severe disease. Surprisingly, among patients with hematological malignancies, either there was no seropositivity or very weak positive antibody response. All other malignancies had similar seropositivity behaviour compared to general population or other co-morbidity like DM, HTN and CAD. Here, it raised the question of comparison of efficacy of immune response between those who had natural immunity after COVID-19 infection without vaccination and those who had acquired immunity after vaccination, for the longevity of antibody persistence and its protective potential. Further studies are required to be conducted on large scale to further elaborate/confirm above findings.
